# Occupational Future Time Perspective Mediates Age Differences in Conflict Strategies

**DOI:** 10.1093/geroni/igaa057.1308

**Published:** 2020-12-16

**Authors:** Dannii Yeung, Alvin Ho

**Affiliations:** City University of Hong Kong, Hong Kong, China

## Abstract

Building on the theoretical framework of socioemotional selectivity theory (Carstensen, 2006), this presentation reports the findings of two studies conducted in Hong Kong Chinese workers to examine whether occupational future time perspective (OFTP) can account for the age differences in conflict strategies. Study 1 is a cross-sectional study with 416 working adults completed an online survey on conflict management (Mage=39.1 years, SD=12.1), and Study 2 is a laboratory study with 123 workers (Mage=40.1 years, SD=12.1) indicated their behavioural responses after watching hypothetical workplace conflict videos. In both studies, five conflict strategies (integrating, compromising, obliging, avoiding, and dominating) and OFTP (focus on opportunities and focus on limitations) were assessed. Parallel mediation analyses were performed. The results of Study 1 showed that both focus on opportunities and focus on limitations mediated the effects of age on obliging (b = -.006, SE=.002; and b = .006, SE=.002, respectively), avoiding (b = -.005, SE=.002; and b =.008, SE=.002, respectively), and dominating (b = -.014, SE=.003; and b = .009, SE=.002, respectively). Focus on opportunities could only account for the effects of age on integrating and compromising. The results of Study 2 showed that only focus on limitation could account for the age variations in the use of avoiding (b = .196, SE = .058) when facing intergenerational conflicts. The findings of this project reveal that the age-related focus on limitations increases older workers’ likelihood to utilize maladaptive conflict strategies, such as dominating and avoiding, to deal with conflicts occurred in the workplace.

